# Human Chitotriosidase Is an Endo-Processive Enzyme

**DOI:** 10.1371/journal.pone.0171042

**Published:** 2017-01-27

**Authors:** Silja Kuusk, Morten Sørlie, Priit Väljamäe

**Affiliations:** 1 Institute of Molecular and Cell Biology, University of Tartu, Tartu, Estonia; 2 Department of Chemistry, Biotechnology and Food Science, Norwegian University of Life Sciences, Ås, Norway; Istituto di Genetica Molecolare, ITALY

## Abstract

Human chitotriosidase (HCHT) is involved in immune response to chitin-containing pathogens in humans. The enzyme is able to degrade chitooligosaccharides as well as crystalline chitin. The catalytic domain of HCHT is connected to the carbohydrate binding module (CBM) through a flexible hinge region. In humans, two active isoforms of HCHT are found–the full length enzyme and its truncated version lacking CBM and the hinge region. The active site architecture of HCHT is reminiscent to that of the reducing-end exo-acting processive chitinase ChiA from bacterium *Serratia marcescens* (*Sm*ChiA). However, the presence of flexible hinge region and occurrence of two active isoforms are reminiscent to that of non-processive endo-chitinase from *S*. *marcescens*, *Sm*ChiC. Although the studies on soluble chitin derivatives suggest the endo-character of HCHT, the mode of action of the enzyme on crystalline chitin is not known. Here, we made a thorough characterization of HCHT in terms of the mode of action, processivity, binding, and rate constants for the catalysis and dissociation using α-chitin as substrate. HCHT efficiently released the end-label from reducing-end labelled chitin and had also high probability (95%) of endo-mode initiation of processive run. These results qualify HCHT as an endo-processive enzyme. Processivity and the rate constant of dissociation of HCHT were found to be in-between those, characteristic to processive exo-enzymes, like *Sm*ChiA and randomly acting non-processive endo-enzymes, like *Sm*ChiC. Apart from increasing the affinity for chitin, CBM had no major effect on kinetic properties of HCHT.

## Introduction

Chitin is an essential structural component of different fungi, nematodes, arthropodes, insects and crustaceans. Chitin is composed of linear β-1-4-linked *N*-acetylglucosamine (NAG) units. The chitin chains are packed into crystals with intervening amorphous regions. In nature chitin is degraded by chitinases, hydrolytic enzymes that are synthesized by different bacteria, fungi and higher eukaryotes. In humans, two chitinases are expressed, chitotriosidase (HCHT) and acidic mammalian chitinase (AMCase) [1,2]. While bacteria degrade chitin for energy supply, human chitinases are parts of innate immune system. Chitin is a good candidate for a non-self molecule recognized by immune system, since there is no chitin synthesized by vertebrates.

HCHT is expressed in macrophages at very low levels in healthy persons [3]. Lately it has been demonstrated that HCHT is also expressed in other cells involved in immune response, such as neutrophils, osteoclasts and Kuppfer cells [4,5]. During the development of acute or chronic inflammatory disorders the enzymatic activity of HCHT increases significantly. Increased activity of HCHT is a biomarker of Gaucher disease [6]. In addition, increased activity of HCHT has been detected in patients with different other diseases, such as sclerosis multiplex, airway hyperresponsiveness and asthma, sarcoidosis, pulmonary tuberculosis and leprosy [7–10]. HCHT regulates the susceptibility to infections of organisms containing chitin as structural component (reviewed in [11–13]). Recombinant HCHT inhibits the hyphal growth of fungi and elevated levels of chitinolytic activity have been detected in blood of guinea pigs infected with *Aspergillus fumigatus* [14,15]. It is proposed, that chitinases degrade chitin-containing structures of pathogens and the released degradation products induce innate immunity.

HCHT is a 50 kDa enzyme belonging to the glycoside hydrolase (GH) family 18 [3]. It consists of two domains–N-terminal 39 kDa catalytic domain and C-terminal carbohydrate binding module (CBM). The two domains are linked with a highly flexible proline rich hinge region of approximately 31 amino acid residues, resulting in a random positioning of CBM relative to the catalytic domain [16]. This is the main difference from well-characterized bacterial chitinases from *S*. *marcescens*, ChiA (*Sm*ChiA) and ChiB (*Sm*ChiB), which display CBMs situated in a clearly defined orientation relative to the catalytic domain, with the two domains connected rigidly to each other [17,18]. About one third of synthesized 50 kDa HCHT is routed to lysosomes and proteolytically cleaved from C-terminus to give a 39 kDa protein that remains catalytically active [19]. In this respect, HCHT resembles to the *S*. *marcescens* endochitinase ChiC (*Sm*ChiC), which is also active in nature in full-length form as well as in C-terminally truncated form without CBM [20].

The catalytic domain of HCHT has an active site cleft homologous to that of *Sm*ChiA with several solvent-exposed aromatic side chains that can stack against the hydrophobic face of the sugars [21]. The cleft contains nine NAG unit binding sites –6 to +3. It is more open than that of *Sm*ChiA being fully extended over one face of the enzyme. This makes HCHT structurally more like an endochitinase, since endoglucanases exhibit shallower active site clefts [22].

The CBM of HCHT belongs, according to the CAZy database, to family CBM14, commonly present in chitinases from *baculoviridae*, invertebrates and mammals [23]. The CBM presents 6 conserved cysteine residues that form S-S bridges essential for maintaining the functional conformation of the domain [24]. In addition, it contains 6 exposed aromatic residues, one of which (Trp465) is conserved and plays a key role in chitin binding [24].

The catalytic activity of HCHT has been characterized on 4-methyl-umbelliferyl and *p*-nitrophenyl-derivatives of NAG oligomers, chitooligosaccharides of different lengths, deacetylated form of chitin–chitosan and β-chitin from squid pen [1,25–29]. From these studies it is known, that HCHT is a chitinase of low processivity (estimates varying between 1.4–11.4 measured on different substrates with different methods) [27,29]. The main hydrolysis product of HCHT is chitobiose (NAG_2_), but similarly to other processive GHs, the product of the first cut can be also NAG [26]. Besides hydrolysis HCHT, can also catalyze transglycosylation reactions [25]. HCHT has been shown to be endo-acting on chitosan [27]. The mode of action on crystalline chitin has not been assessed. Also, the directionality of HCHT has not been determined. The thermodynamic signature of the binding of the potent inhibitor, allosamidin to HCHT is similar to that of *Sm*ChiA, indicating that the hydrolysis directionality of the two enzymes may be the same [30, 31]. However, in the crystal structures of HCHT with NAG_2_, the NAG_2_ is found in subsites –2/–1 [21]. Strong binding of dimers to product binding sites (+1 and +2 in case of *Sm*ChiA) has been shown for other processive GHs and such strong product binding has been shown to drive the processive movement of the enzymes [32–35]. Thus, the question of the directionality of HCHT remains still open.

Here we made a thorough characterization of HCHT in terms of the mode of action, processivity, binding, and rate constants for the catalysis and dissociation using α-chitin as substrate. Both HCHT isoforms 50 kDa full-length protein (HCHT50) and its 39 kDa truncated version without the C-terminal CBM (HCHT39) were included into the study. The kinetic properties of HCHT were found to be in-between those characteristic to processive exo-enzymes, like *Sm*ChiA, and randomly acting non-processive endo-enzymes, like *Sm*ChiC.

## Materials and Methods

### Materials

Crab chitin (Sigma C7170), NAG_2_ (Sigma D1523), chitosan, 4-methyl-umbelliferyl-β-diacetylchitobioside hydrate (MU-NAG_2_) (Sigma M9763), anthranilic acid (AA), sodium cyano-borohydride, sodium borohydride (NaBH_4_), 3-methyl-2-benzo-thiazoline hydrazine hydrochloride hydrate (MBTH) and bovine serum albumin (BSA) were purchased from Sigma-Aldrich. All chemicals were used as purchased.

### Enzymes

Protein expression and purification of the two isoforms of HCHT is described in Stockinger *et al*. [29]. In brief, proteins were produced in HEK293-6E cells. Two vectors were constructed designated pHCHT50 and pHCHT39 expressing HCHT including its native signal peptide and with and without the C-terminal chitin binding domain, respectively. The genes were synthesized (Genescript) as BamHI-XbaI fragments and ligated into pTT5V5H8Q (NRC Biotechnology Research Institute) resulting in a C-terminal His-tag (8xHis) on the recombinant proteins produced. The cloning steps were performed in *Escherichia coli* DH5α. pHCHT50 and pHCHT39 were transfected into HEK293-6E cells grown in F17 medium (Invitrogen) supplemented with Kolliphor P188 (Sigma) and L-glutamate (Sigma) to final concentrations of 0.1% and 4 mM, respectively. Cultivation of cells took place in 90 mL medium in disposable 500 mL flasks with gentle shaking (70 rpm, at 37°C, with 5% CO_2_ and 80% humidity). Transfection was performed with PEIpro (Polyplus) when the cell density in the cultures was 1.7 × 10^6^ cells/mL. Tryptone N1 feeding medium (TekniScience) was added to a final concentration of 0.5% 48 h after transfection, and harvesting of the protein containing culture supernatant was performed 96 h after transfection. Harvesting took place at a cell density of 2.2 × 10^6^ cells/mL. Recombinant HCHT was purified using a HisTrap HP column (GE Healthcare) according to the instructions given by the manufacturer.

**Fig 1:**
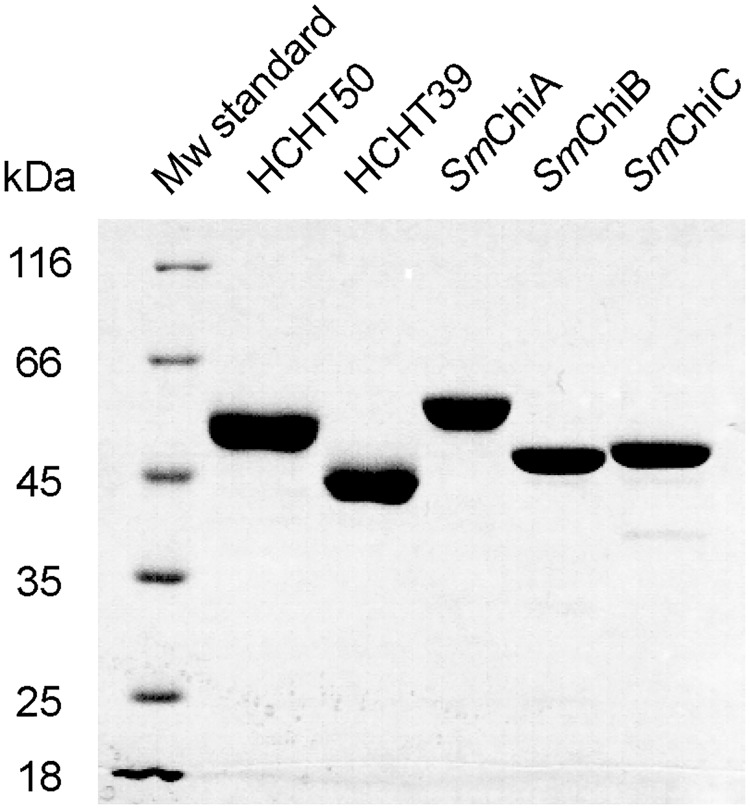
SDS-PAGE analysis of purified chitinases. 4–6 μg purified HCHT 50 kDa and 39 kDa isoforms and *S*. *marcescens* chitinases *Sm*ChiA, *Sm*ChiB and *Sm*ChiC were loaded and the gel was stained with Coomassie Brilliant Blue G-250.

Protein expression and purification of *Sm*ChiA and *Sm*ChiB is described in Brurberg *et al*. [36]. Protein expression and purification of *Sm*ChiC is described in Synstad *et al*. [37]. The purity of all enzymes was > 95% as judged by SDS-PAGE (Fig 1). Enzymes concentrations were determined by using the Bradford method from BioRad.

### Chitin substrates

Crystalline α-chitin was prepared of crab chitin as described in Kurashin *et al*. [35]. Briefly, crude crab chitin was suspended in water, incubated in 0.55 M HCl for 2 h at room temperature with 3 changes, washed three times with water, incubated with 0.3 M NaOH at 70°C for 3 h with 3 changes, followed by several washes with water. Next, the chitin was washed with ethanol and incubated in acetone for 1 h with 2 changes at room temperature, washed repeatedly with water and grinded in mortar. To ensure that all amino-groups were acetylated the purified chitin was treated with acetic anhydride. For that the chitin was washed three times with methanol and finally re-suspended in methanol to give the concentration of 20 mg/mL. 1 mL of acetic anhydride was added per 1 g of chitin and the mixture was incubated overnight at room temperature, with stirring. Next, O-deacetylation was carried out by adding 100 mM KOH in methanol and incubating for 4 h at room temperature, with stirring. After that the chitin was washed repeatedly with water and 50 mM sodium acetate, pH 6.1. Finally 0.01% NaN_3_ was added and the chitin was stored at 4°C.

Chitin nanowhiskers (CNWs) and ^14^C labelled CNWs (^14^C-CNWs) were prepared as described in Kuusk *et al*. [38]. A total of 35 g of crab chitin was treated with HCl, NaOH, ethanol and acetone as described above. The purified chitin was suspended in 3 M HCl and incubated at 100°C for 90 min, with stirring. The mixture was diluted 3-fold with water, neutralized by slowly adding solid NaOH and finally buffered with 10 mM sodium acetate, pH 6.1. CNWs were washed several times with 10 mM sodium acetate, pH 6.1, and stored at 4°C. The N-acetylation and O-deacetylation of CNWs were carried out essentially as described for crystalline α-chitin (see above). To prepare ^14^C-CNWs the N-acetylation of CNWs was carried out with 5 mCi of [1-^14^C]acetic anhydride as described in Kuusk *et al*. [38]. The specific radioactivity of ^14^C-CNWs was 4.18 × 10^6^ dpm/mg.

Amorphous chitin was prepared by the acetylation of chitosan as described in Kurashin *et al*. [35]. Chitosan was suspended in water, an equal volume of 20% acetic acid was added, with stirring, and the mixture was diluted five times by adding methanol, with stirring. 1 mL of acetic anhydride was added per 1 g of chitin, with stirring, and the mixture was incubated overnight at room temperature, without stirring. Next, the mixture was diluted further five times with water. The acetic acid was neutralized and the O-deacetylation was carried out by adding NaOH to the final concentration of 50 mM, followed by incubating overnight at room temperature, with stirring. The amorphous chitin was repeatedly washed with water and 50 mM sodium acetate, pH 6.1. Finally 0.01% NaN_3_ was added and the chitin was stored at 4°C.

Reduced chitin was prepared of α-chitin by NaBH_4_ treatment as described in Kurashin *et al*. [35]. The purified α-chitin was washed twice with 0.25 M NaHCO_3_/Na_2_CO_3_ pH 10 and resuspended in the same buffer to give the chitin concentration 2 mg/mL. The mixture was heated to 80°C, 5 M sodium borohydride in 0.1 M NaOH was added to give the final concentration of sodium borohydride of 25 mM, followed by 1 h of incubation. The same amount of 5 M sodium borohydride in 0.1 M NaOH was added for four more times with 1 h of incubation at 80°C after each. To quench the reaction, equal volume of 0.5 M acetic acid was added and the mixture was incubated overnight at room temperature, with stirring. The reduced chitin was washed repeatedly with water and 50 mM sodium acetate, pH 6.1, 0.01% NaN_3_ was added and the chitin was stored at 4°C.

Reducing-end AA-labeled crystalline α-chitin (AA-α-chitin) was prepared as described in Kurashin *et al*. [35]. For reducing-end AA-labeling of CNWs the CNWs in 50 mM sodium acetate pH 6.1 were added to buffered methanol until the methanol concentration was 80%. Sodium cyanoborohydride and AA were added to the concentrations of 0.5 M and 50 mM, respectively. The reaction was carried out for 2 h at 80°C. The AA-labelled CNWs were washed repeatedly with 47.86 mM NaOH through centrifugation (5 min at 4000×g) and re-suspension steps. Finally, 20% glacial acetic acid was added to adjust pH to 6.1, and AA-CNWs were stored at 4°C.

### Determining the directionality of hydrolysis

AA-CNWs (1 mg/mL) were incubated with *Sm*ChiA (10 nM), *Sm*ChiB (10 nM), HCHT50 (10 nM), HCHT39 (10, 20 or 50 nM) or *Sm*ChiC (100 nM) in 50 mM sodium acetate, pH 6.1, supplemented with BSA (0.1 mg/mL) at 37°C, with stirring. After 5, 10, 20, 40 and 60 min aliquots were taken out, the reaction was stopped by adding NaOH to 0.2 M, and the chitin was pelleted by centrifugation (2 min at 10^4^×g). The concentration of soluble AA-sugars was determined by measuring the fluorescence in the supernatant using excitation and emission wavelengths set to 330 nm and 425 nm, respectively. The relative fluorescence of 450 intensity units/μM measured for AA-labeled NAG was used for calibration [35]. The concentration of the reducing groups in the supernatant was measured using the MBTH method [39]. The degree of total degradation of AA-CNWs (in %) was calculated from the released reducing groups assuming NAG_2_ as the sole hydrolysis product. This assumption is plausible since NAG_2_ is the predominant product (> 90%) in hydrolysis of crystalline chitin by *S*. *marcescens* chitinases [40] as well as HCHT [29]. The degree of the released reducing-end-label (in %) was calculated from the released AA-sugars and the total amount of AA-label in AA-CNWs (9.3 ± 0.4 μmol/g).

### Measuring apparent processivity (P^app^)

Reduced chitin (1 mg/mL) was incubated with 10 nM HCHT50, HCHT39 or 1 nM *Sm*ChiC in 50 mM sodium acetate, pH 6.1, supplemented with BSA (0.1 mg/mL) at 37°C, with stirring. At defined times, aliquots were withdrawn and the reaction was stopped by adding NaOH to 0.2 M. The chitin was pelleted by centrifugation (5 min at 10^4^×g) and the amount of soluble reducing groups (SRGs) in the supernatant was measured using the MBTH method. The amount of enzyme generated insoluble reducing groups (IRGs) was determined by fluorescence labeling of the enzyme treated reduced chitin with AA as described in Kurashin *et al*. [35]. Briefly, the chitin pellet was washed twice with water, once with 50 mM sodium acetate, pH 6.1, and twice with water. The chitin was re-suspended in 200 μL water and the AA labeling was carried out in 80% buffered methanol with 0.5 M sodium cyanoborohydride and 50 mM AA at 80°C for 2 h. The AA-labeled chitin was washed three times with water and three times with 50 mM sodium acetate, pH 6.1. Finally, the chitin was re-suspended in 50 mM sodium acetate, pH 6.1 to the final concentration of 0.5 mg/mL and the fluorescence of the suspension was measured using excitation and emission wavelengths set to 330 nm and 425 nm, respectively. Relative fluorescence of 310 intensity units/μM determined for AA-labeled NAG in 0.5 mg/mL chitin suspension was used for calibration [35]. The value of P^app^ was found as a slope of the linear regression line of the data plotted in coordinates of ([IRG] + [SRG])/[IRG] [35,41,42].

### Measuring the probability of endo-mode initiation (P_endo_)

Before using, AA-α-chitin was treated with NaOH to remove nonspecific label. For that the AA-α-chitin was incubated in 0.2 M NaOH for 15 min at room temperature, followed by three washes with 50 mM sodium acetate, pH 6.1. Washed AA-α-chitin (1 mg/mL) was incubated with 10 nM HCHT50 or HCHT39 in 50 mM sodium acetate, pH 6.1, supplemented with BSA (0.1 mg/mL) at 37°C, with stirring. At defined times, the reaction was stopped by adding NaOH to 0.2 M and the chitin was pelleted by centrifugation (2 min at 10^4^×g). The concentration of the reducing groups in the supernatant was measured using the MBTH method. The concentration of soluble AA-sugars was determined by measuring the fluorescence in the supernatant using excitation and emission wavelengths set to 330 nm and 425 nm, respectively. The relative fluorescence of 450 intensity units/μM measured for AA-labeled NAG was used for calibration [35]. The number of AA-sugars released from AA-α-chitin ([AA-sugars]) was taken equal to the number of exo-initiations from the reducing-end. The sum of the numbers of reducing-end exo-initiations and endo-initiations was taken equal to the number of IRGs ([IRG]) generated to the reduced α-chitin under exactly the same experiment conditions (see measuring P^app^ above). The P_endo_ was calculated according to P_endo_ = ([IRG]–[AA-sugars])/[IRG] [35,41].

### Measuring initial rates

Crystalline α-chitin, CNWs, or amorphous chitin (0.1–10 mg/mL) were incubated with 100 nM HCHT50 or HCHT39 in 50 mM sodium acetate, pH 6.1, supplemented with BSA (0.1 mg/mL) at 37°C for 1 min, without stirring. The reaction was stopped by adding NaOH up to 0.2 M. For t = 0, NaOH was added before the enzyme. Chitin was sedimented by centrifugation (5 min at 10^4^×g) and the concentration of the reducing groups in the supernatant was measured using the MBTH method. In the case of CNWs as substrate an additional amount of CNWs (to 3 mg/mL) were added to the NaOH stopped reactions before centrifugation. This was done in order to improve the sedimentation of CNWs during centrifugation [38].

### Measuring the total binding of HCHT to crystalline α-chitin ([HCHT_bound_])

Crystalline α-chitin (1 mg/mL) was incubated with 10 nM HCHT50 or HCHT39 in 50 mM sodium acetate, pH 6.1, supplemented with BSA (0.1 mg/mL) at 37°C, with stirring. At selected times, 400 μL aliquots were withdrawn and chitin was sedimented by centrifugation (1 min 10^4^×g). The concentration of the free enzyme in the supernatant ([HCHT_free_]) was assesseded by measuring the MU-NAG_2_ hydrolyzing activity in the supernatant. For that 100 μL of the supernatant was added to 100 μL of 10 μM MU-NAG_2_ and incubated for 2 min at 37°C, without stirring. The reactions were stopped by adding NaOH to 10 mM. The concentration of MU ([MU]) was quantified by fluorescence with excitation and emission wavelengths set to 360 nm and 450 nm, respectively. Before fluorescence measurements the volumes of the reaction mixtures were brought to 1 mL with 0.1 M ammonium hydroxide. [HCHT_free_] was found from the released [MU] using the calibration curves made for the hydrolysis of MU-NAG_2_ (5 μM) at different HCHT concentrations (0.5–10 nM). Concentration of HCHT bound to chitin ([HCHT_bound_]) was found as a difference between the total concentration of HCHT and [HCHT_free_].

### Measuring the concentration of HCHT with active site occupied by the chitin ([HCHT_bound-OA_])

[HCHT_bound-OA_] was measured by following the inhibition of MU-NAG_2_ hydrolyzing activity of HCHT by crystalline α-chitin [38]. α-chitin (1 mg/mL) was incubated with 10 nM HCHT50 or HCHT39 in 50 mM sodium acetate, pH 6.1, supplemented with BSA (0.1 mg/mL) at 37°C, with stirring. At defined times 100 μL aliquots of reaction mixtures were pipetted to 100 μL of 10 μM MU-NAG_2_ and incubated for 2 min at 37°C, without stirring. The reactions were stopped by adding NaOH to 10 mM and the volumes of the reaction mixtures were brought to 1 mL with 0.1 M ammonium hydroxide. Chitin was pelleted by centrifugation (1 min 10^4^×g) and the concentration of MU in the supernatant was quantified by fluorescence (see above). The concentration of HCHT with free active sites ([HCHT_FA_]) was found from the rates of MU-NAG_2_ hydrolysis in the presence of crystalline α-chitin using the calibration curves made without chitin. The possible inhibition of MU-NAG_2_ hydrolyzing activity of HCHT by NAG_2_ released from chitin was judged to be negligible. [HCHT_bound-OA_] was found as a difference between the total concentration of HCHT and [HCHT_FA_].

### Hydrolysis of MU-NAG_2_ and inhibition with NAG_2_

MU-NAG_2_ (1–50 μM) was incubated with 1 nM HCHT50 or HCHT39 in 50 mM sodium acetate pH 6.1 supplemented with BSA (0.1 mg/mL) at 37°C, without stirring. At defined times the reactions were stopped by adding NaOH to 10 mM and the volumes were brought to 1 mL with 0.1 M ammonium hydroxide. The concentration of released MU was determined by fluorescence with excitation and emission wavelengths set to 360 nm and 450 nm, respectively. For zero time points NaOH was added before the enzyme. Inhibition of MU-NAG_2_ hydrolysis by NAG_2_ was studied at three different MU-NAG_2_ concentrations (0.5, 5.0 and 50 μM) by varying the concentration of NAG_2_ between 0.05–2 μM. NAG_2_ inhibition of the hydrolysis of chitin was assessed using ^14^C-CNWs as substrate. For that, ^14^C-CNWs (1.0 or 5.0 mg/mL) were incubated with 1 nM HCHT50 or HCHT39 in 50 mM sodium acetate pH 6.1 supplemented with BSA (0.1 mg/mL) at 37°C, without stirring. A defined amount of NAG_2_ was added to the reactions. The reaction was stopped at selected times by adding NaOH to 0.2 M. Non-labeled CNWs (to 3 mg/mL) were added and chitin was separated by centrifugation (5 min 10^4^×g) and the amount of radioactivity in the supernatant was quantified using a liquid scintillation counter. [38].

## Results

### HCHT is efficient in releasing the reducing-end label from crystalline chitin

**Fig 2:**
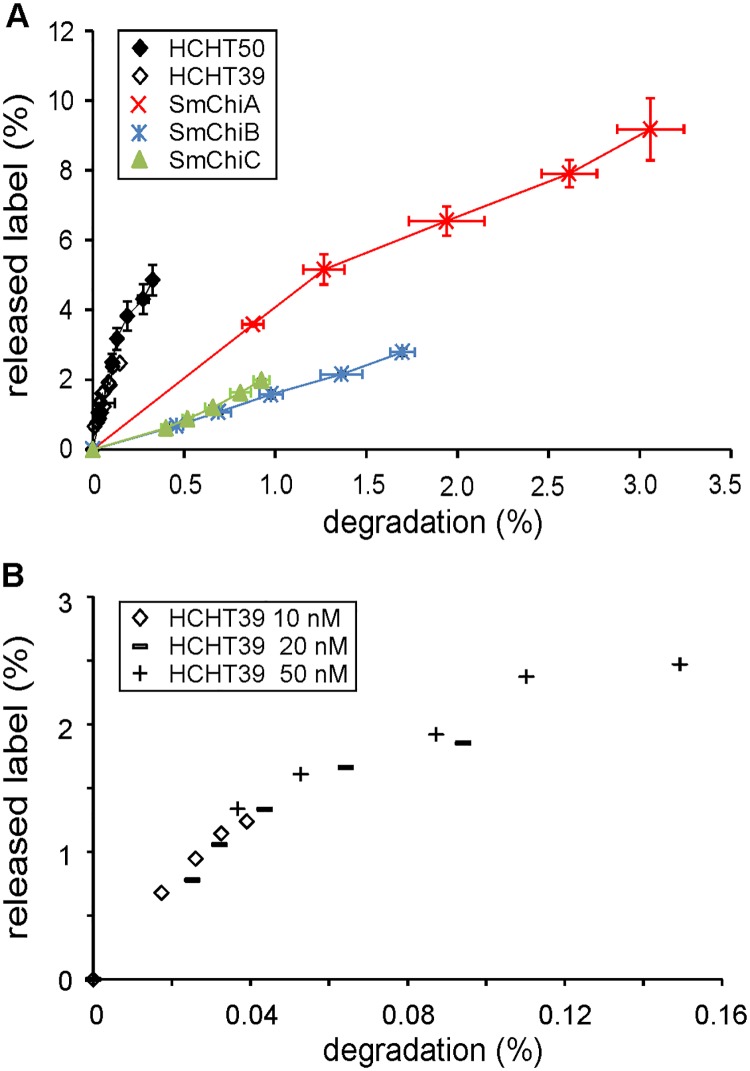
Progress curves of AA-CNW hydrolysis. (A) AA-CNWs (1 mg/mL) were hydrolysed with HCHT50, HCHT39, *Sm*ChiA, *Sm*ChiB or *Sm*ChiC at 37°C. The release of AA-labelled sugars and total soluble reducing ends were measured at defined time points (5, 10, 20, 40 and 60 min). Error bars show standard deviations and are from three independent experiments. (B) Progress curves at different concentrations of HCHT39.

According to its active site architecture, HCHT is reminiscent to *Sm*ChiA, a processive enzyme moving towards the non-reducing end of chitin chain [43,44]. However, the experimental evidence for the directionality in the degradation of chitin polymer is lacking. As demonstrated with cellulases, the directionality of hydrolysis can be assessed upon the hydrolysis of reducing-end labeled polymeric substrates [45,46]. When plotted in coordinates released end-label (%) *versus* total degradation (%), the hydrolysis by reducing-end exo-acting enzymes results in convex curves whereas non-reducing-end exo-active enzymes generate concave progress curves [45,46]. Here, we followed the hydrolysis of reducing-end anthranilic acid (AA) labeled CNWs (AA-CNWs) by full-length HCHT and its 39 kDa isoform lacking the CBM. The *S*. *marcescens* chitinases with well characterized mode of action were also included. Most effective in releasing AA-label from AA-CNWs was HCHT followed by *Sm*ChiA (Fig 2A). Convex progress curves with high initial slopes are indicative of reducing-endo exo-mode initiations with low processivity [45]. Hydrolysis by both isoforms of HCHT resulted in overlapping convex progress curves indicating that CBM has no role in determining the mode of action of the enzyme. Experiments made at different concentrations of HCHT39 resulted in overlapping progress curves (Fig 2B) indicating that the mode of action is independent of enzyme concentration. In accord with its opposite directionality, *Sm*ChiB was much less efficient in releasing the reducing-end label from AA-CNWs compared to *Sm*ChiA. The progress curve of endochitinase *Sm*ChiC was similar to that of *Sm*ChiB. The use of endo-mode initiation obviously complicates the interpretation of progress curves [45]. Totally random mode of initiation with low processivity is expected to result in the equal extent of the release of end-label and total degradation [45]. However, this implies uniform distribution of the end-label, which is not possible to achieve in practice with crystalline substrates. This is because the chain ends buried inside the chitin crystal are not accessible for labeling. As a result, the relative amount of AA label in outer layers of chitin crystal is higher than that in inner layers [46].

### HCHT preferentially uses endo-mode initiation in hydrolysis of α-chitin

**Fig 3:**
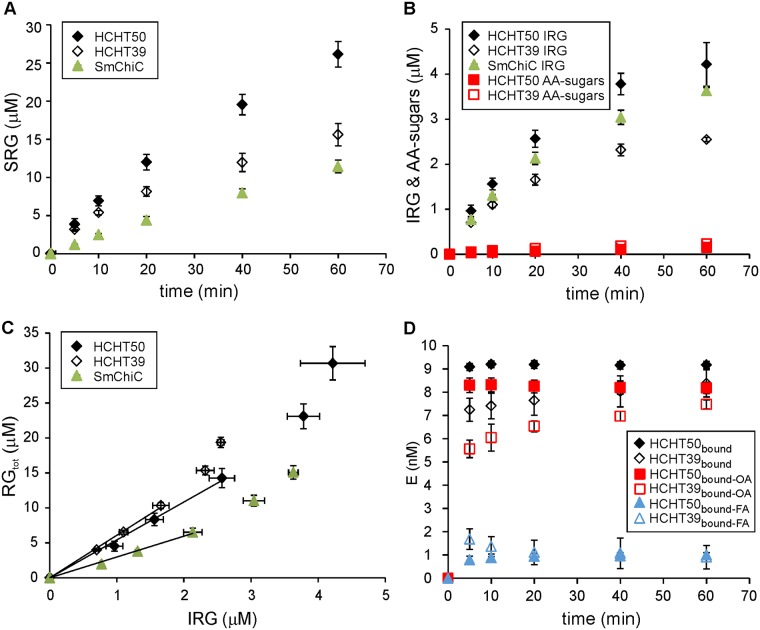
Processivity and probability of endo initiation of HCHTs. AA-α-chitin (1 mg/mL) or reduced α-chitin (1 mg/mL) were hydrolyzed with HCHT50, HCHT39 (10 nM) or *Sm*ChiC (1 nM) at 37°C. (A) The release of soluble reducing groups (SRGs). Shown are the combined results with AA-α-chitin and reduced α-chitin. Error bars are from six independent experiments, three made with AA-α-chitin and three with reduced α-chitin as substrate. (B) The release of AA-labelled sugars from AA-α-chitin and the formation of insoluble reducing groups (IRG) on reduced α-chitin under otherwise identical conditions. (C) Data of the hydrolysis of reduced α-chitin from panels (A) and (B) plotted in the coordinates of total reducing groups (RG_tot_ = IRG + SRG) *versus* IRG. The solid lines represent the best fit of linear regression (only the data points shown within the solid lines were included in linear regression analysis). The slope of the solid line from linear regression equals to apparent processivity, P^app^. (D) Discrimination between different populations of HCHT bound to α-chitin. The total concentration of HCHT was 10 nM and that of α-chitin was 1 mg/mL. The concentration of total bound HCHT ([HCHT]_bound_) was found as a difference between the total concentration of the enzyme and the concentration of the enzyme free in solution. The concentration of HCHT with free active site was measured by following the MU-NAG_2_ hydrolyzing activity of HCHT in the presence of α-chitin. The concentration of bound HCHT with active site occupied by chitin ([HCHT] _bound OA_) was found as a difference between the total concentration of the enzyme and that with free active site. The concentration of bound HCHT with free active site ([HCHT] _bound FA_) was found as a difference between the [HCHT]_bound_ and [HCHT] _bound OA_. Error bars show standard deviations and are from three independent experiments.

It has been shown previously that HCHT acts as an endo enzyme on soluble substrates such as chitosans [27–29]. However, the soluble nature of chitosan may promote the use of the endo-mode of action of an enzyme [27]. Therefore, we tested the possible use of endo-mode initiation on insoluble α-chitin. For this we prepared two different substrates: one with a fluorescence label at the reducing ends of chitin chains–reducing-end AA-labeled α-chitin (AA-α-chitin), and the other with the reducing ends reduced with NaBH_4_ to corresponding alditols–reduced α-chitin. These two substrates were used in parallel hydrolysis experiments with HCHT in identical reaction conditions. We measured the release of AA-sugars from AA-α-chitin and compared it with the number of insoluble reducing groups (IRGs) generated in the hydrolysis of reduced α-chitin (Fig 3B). The release of soluble reducing groups (SRGs) from both substrates was identical (Fig 3A), indicating that the general activity of the enzyme was not affected by the nature of the reducing end of α-chitin. The number of released AA-sugars from AA-α-chitin represents the number of initiations from the reducing end. The number of IRGs generated to reduced α-chitin represents the sum of the endo-mode initiations and the initiations from the reducing end. Therefore, the probability of endo mode initiation (relative to that of reducing-end exo) can be found as P_endo_ = ([IRG]–[AA-sugars]) / [IRG] [35,41]. Both HCHT variants had P_endo_ values around 0.95, indicating the predominant use of endo-mode initiation on crystalline α-chitin (Table 1).

**Table 1:** Endo-probability and processivities measured on α-chitin.

Enzyme	P_endo_	P^app^	*k*_IRG_* (1/s)	P^intr^*
HCHT50	0.95±0.1	5.6±0.9	0.39±0.05**	8.6±1.1
HCHT39	0.95±0.2	6.3±0.1	0.42±0.02**	8.9±0.4
*Sm*ChiC	nd	3.0±0.2	2.57±0.07***	nd

nd, not determined

* These parameters were calculated using 5 min reaction time.

** Calculated using the equation *k*_IRG_ = *v*_IRG_ / [E]_bound OA_.

*** Calculated using the equation *k*_IRG_ = *v*_IRG_ / [E]_total_.

### HCHT shows limited processivity in hydrolysis of α-chitin

Apparent processivity of an enzyme (P^app^) is the experimentally measured value of processivity on a real polymer. P^app^ is defined as the number of catalytic events divided by the number of the initiations of processive runs. It has been shown that, for the GHs employing reducing-end-exo and/or endo-mode initiation, P^app^ can be found from the hydrolysis of a reduced polymeric substrate under single-hit conditions (*i*.*e*. in the conditions that minimize the chance of the same chain being hit twice) [41,42]. The number of catalytic events is represented by the sum of soluble reducing ends (SRGs) and insoluble reducing ends generated to reduced polymeric substrate (IRGs). For an enzyme employing reducing-end exo- and/or endo-mode initiation, the number of the initiations of processive runs equals to the number of IRGs generated on reduced chitin. Thus, P^app^ of HCHT can be calculated using the equation P^app^ = (IRG + SRG) / IRG. Here we followed the hydrolysis of reduced α-chitin by both variants of HCHT. *Sm*ChiC was studied in parallel. The most efficient in generating IRGs was *Sm*ChiC followed by HCHT50 and HCHT39 (Fig 3B). The same trends were observed also in the release of SRGs (Fig 3A, note that the concentration of HCHT was 10-fold higher than that of *Sm*ChiC). The value of P^app^ can be found as the slope of the linear regression line of the data plotted in coordinates of (IRG + SRG) *versus* IRG (Fig 3C). With all enzymes a slight deviation from linearity was observed at higher IRG concentrations, indicating a deviation from single-hit conditions at longer hydrolysis times. Therefore, only the data points within the linear regions of curves were used in calculating the P^app^ values. Both HCHT isoforms displayed limited processivity, which was still about twofold higher than that measured for *Sm*ChiC (Table 1).

### Binding of HCHT to chitin–differentiation between different populations of bound enzyme

Binding of enzymes to insoluble polysaccharides is often assessed by measuring the concentration of enzyme free in solution. For that, the polysaccharide bound enzyme is separated from that free in solution by centrifugation or filtration. The concentration of total bound enzyme can be found as a difference between total enzyme concentration and that free in solution. However, more detailed information about the binding is available from the experiments, where the hydrolysis of a low molecular weight reporter molecule is performed in the presence of polymeric substrate of interest [38,47–50]. Only the enzyme molecules with active site free from polymer can hydrolyze the reporter molecule and this forms the basis for quantification of the population of enzymes with free active site. The concentration of bound enzyme with active site occupied by the polymer ([E]_bound-OA_) can now be found as a difference between the total concentration of the enzyme and that with free active site. Furthermore, when the concentration of total bound enzyme ([E]_bound_) is measured in parallel, the concentration of bound enzyme with free active site ([E]_bound-FA_) can be found as a difference between [E]_bound_ and [E]_bound-OA_. Here we assessed the kinetics of the binding of HCHT (10 nM) to α-chitin (1 mg/mL). MU-NAG_2_ was used as a reporter molecule in measuring the active site mediated binding. For the full-length HCHT, the concentrations of all populations of bound enzyme were at their plateau value after the first 5 min of incubation with α-chitin (Fig 3D). The binding of HCHT39 was somewhat slower than that of HCHT50, especially at the level of the active site mediated binding (Fig 3D). It is noteworthy that both isoforms had a significant population of bound enzyme with free active site (Fig 3D). However, we note as a caveat here that there is a possibility that HCHT is bound to chitin through the substrate binding region of active site cleft so that subsites -2 to +1 are available for the hydrolysis of MU-NAG_2_. This binding mode reveals as [HCHT]_bound-FA_ and not as [E]_bound-OA_ in our experiment.

### Dissociation rate constant of HCHT

Like P^app^, the dissociation rate constant (*k*_*off*_) of GHs employing reducing-end-exo and/or endo-mode initiation can be found from the hydrolysis of a reduced polymeric substrate under single-hit conditions [41,42]. It has been shown that, when calculated as *k*_*IRG*_ = *v*_*IRG*_ / [E]_bound-OA_, the rate constant of the formation of IRGs (*k*_*IRG*_) represents *k*_*off*_ [47]. In this study, *v*_IRG_ is the rate of the generation of IRGs (*v*_IRG_) upon the hydrolysis of reduced chitin and [E]_bound-OA_ stands for the concentration of bound HCHT with active site occupied by chitin (see above). *Sm*ChiC was studied in parallel with both HCHT isoforms. With all enzymes studied, *v*_IRG_ decreased with hydrolysis time as evidenced by non-linear time curves of IRG formation (Fig 3B). This non-linearity is common in hydrolysis of recalcitrant polysaccharides and is apparently caused by changes in substrate with hydrolysis time [51]. Therefore, we estimated the *k*_off_ value using the rate of IRG formation and [E]_bound-OA_ after the first 5 min of hydrolysis (Fig 3B & 3D). Both HCHT isoforms displayed similar *k*_off_ values (Table 1) with half-lives around 1.7 s. Unfortunately there is no suitable reporter molecule for measuring the active site mediated binding of *Sm*ChiC. Using the total enzyme concentration instead of [E]_bound-OA_ provides a minimum estimate for the *k*_off_ value of *Sm*ChiC, which was still about an order of magnitude higher than the *k*_off_ measured for HCHT (Table 1).

### Catalytic constant and intrinsic processivity of HCHT

**Fig 4:**
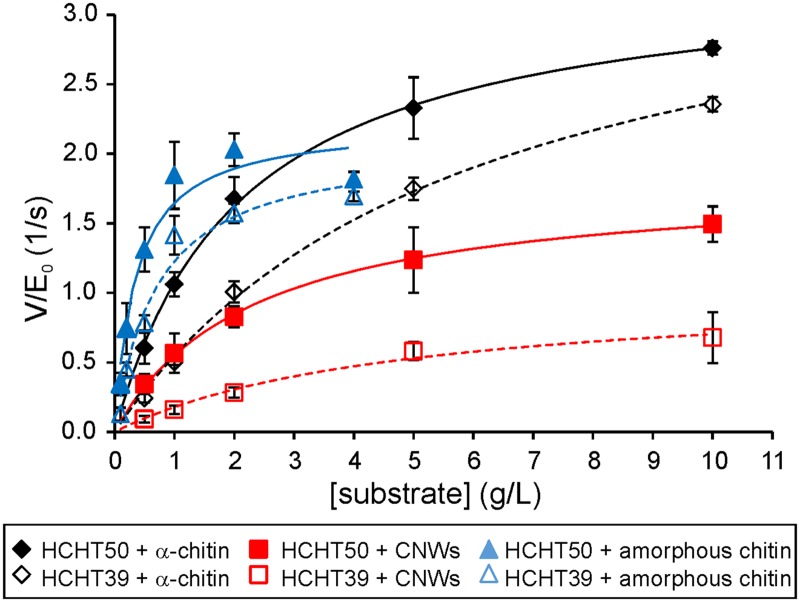
Michaelis-Menten kinetics of HCHTs. α-chitin, amorphous chitin or CNWs were hydrolyzed with HCHT50 or HCHT39 at 37°C for 1 min. The solid lines represent the best fit according to the Michaelis-Menten equation. Error bars show standard deviations and are from three independent experiments.

An important kinetic parameter of polymer-active enzymes is the enzymes intrinsic processivity (P^Intr^) [41,42]. While P^app^ is a processivity value measured under given experiment conditions, P^Intr^ represents the processivity potential of an enzyme. P^Intr^ is governed by catalytic constant (*k*_cat_) and *k*_off_, and for the processive enzymes P^Intr^ can be approximated by P^Intr^ ≈ *k*_cat_ / *k*_off_ [41,42,52] The estimates for *k*_cat_ can be found from the measurements of initial rates at saturating substrate concentrations as *k*_cat_ = *V*_max_ / [E]_total_. Here we measured the release of SRGs by HCHT after 1 min of hydrolysis on 3 different substrates– α-chitin, CNWs, and amorphous chitin. With all enzymes and substrates, the initial rates measured at different substrate concentrations followed the Michaelis-Menten saturation kinetics (Fig 4). Resulting *V*_max_/[E]_total_ and *K*_m_ values are listed in Table 2. The two HCHT isoforms had similar *V*_max_/[E]_total_ values though the full length enzyme had about two-fold lower *K*_m_ values. This points to the contribution of CBM in chitin binding. Comparing the action of enzymes on different substrates shows that the *K*_m_ values of both HCHT isoforms on amorphous chitin were an order of magnitude lower than corresponding figures on crystalline substrates, α-chitin and CNWs. The differences in *V*_max_/[E]_total_ values on different substrates were less prominent (Table 2). Provided with *V*_max_/[E]_total_ values as estimates of *k*_cat_, we can now calculate the P^Intr^ values on α-chitin using the value of *k*_IRG_ measured with reduced α-chitin as an estimate of *k*_off_ (Table 1). The P^Intr^ values of both isoforms were similar to each other within error limits. Although the P^Intr^ values were somewhat higher than P^app^ values measured on reduced α-chitin, the difference was far less prominent than that usually found for processive GHs [35,41].

**Table 2:** Michaelis-Menten kinetic parameter values on different chitin substrates.

	HCHT50		HCHT39	
Substrate	*K*_m_ (g/L)	*V*_max_/E_0_ (1/s)	*K*_m_ (g/L)	*V*_max_/E_0_ (1/s)
amorphous chitin	0.35±0.05	2.20±0.10	0.70±0.19	2.09±0.20
α-chitin	2.11±0.2	3.34±0.11	5.84±0.05	3.75±0.06
CNWs	2.30±0.28	1.82±0.07	4.81±0.93	1.04±0.09

### Product inhibition of HCHT

**Fig 5:**
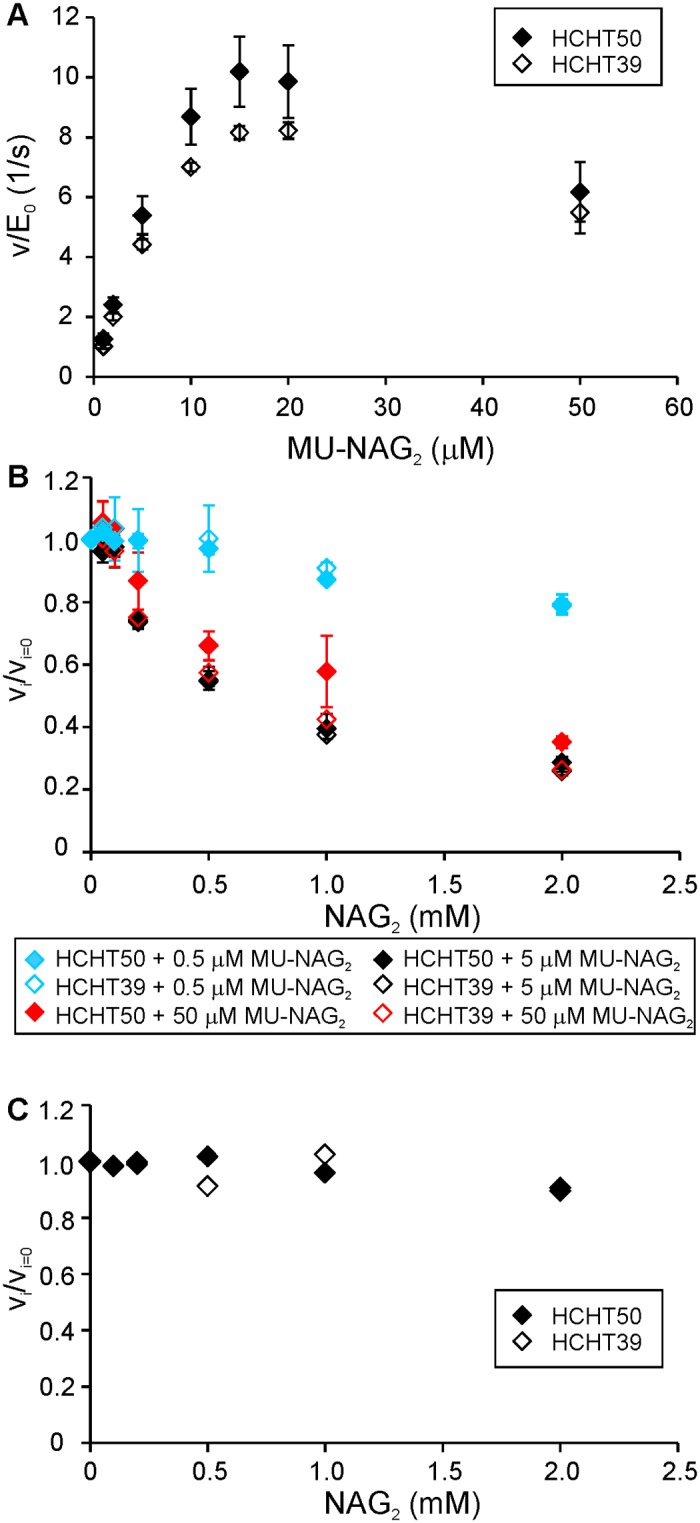
NAG_2_ inhibition of HCHT. (A) Activity of HCHT on MU-NAG_2_ substrate as a function of substrate concentration. (B) NAG_2_ inhibition of HCHT on MU-NAG_2_ substrate measured at 3 different substrate concentrations—0.5 μM, 5 μM or 50 μM. (C) NAG_2_ inhibition of HCHT on ^14^C-CNW substrate (1.0 g/L). *v*_i_ and *v*_i = 0_ stand for the rates measured in the presence and absence of inhibitor, respectively. Error bars show standard deviations and are from three independent experiments.

Strong binding of disaccharide product to product binding subsites is required for processivity [34,38,53]. Therefore, processive GHs are more sensitive to product inhibition than their non-processive counterparts. Here we studied the NAG_2_ inhibition of HCHT in the hydrolysis of low-molecular weight model substrate, MU-NAG_2_, as well as crystalline chitin (^14^C-CNW). Inconsistent with Michaelis-Menten saturation kinetics, the release of MU from MU-NAG_2_ showed distinct substrate inhibition at higher MU-NAG_2_ concentrations (Fig 5A). The phenomenon of substrate inhibition is often reported with retaining GHs and can be accounted for by transglycosylation to substrate [25,54,55]. Breakdown of the Michaelis-Menten kinetics did not permit to assess the effects of inhibitor to *V*_max_ and *K*_m_. Inhibition of MU-NAG_2_ hydrolysis by NAG_2_ was studied at substrate concentrations below (0.5 μM, and 5 μM) and above (50 μM) the optimal substrate concentration (around 15–20 μM). The NAG_2_ inhibition appeared weaker in the case of 0.5 μM substrate concentration and was nearly equal in the case of 5 μM and 50 μM MU-NAG_2_ concentrations. This pattern is reminiscent to uncompetitive inhibition, a mechanism of inhibition where the inhibitor binds to the enzyme-substrate complex but not to the free enzyme. Furthermore, the NAG_2_ inhibition of HCHT was different from the “conventional” hyperbolic decrease of rate with increasing inhibitor concentration (Fig 5B). The enzyme appeared to be slightly activated at low NAG_2_ concentrations followed by inhibition at higher concentrations. Such an apparent activation by inhibitor is often observed with glucose tolerant β-glucosidases. Mechanistic interpretations of the phenomenon include transglycosylation reactions to inhibitor but also the competition of inhibitor with the non-productive binding of substrate [56–58]. A more detailed analysis of the inhibition mechanisms of HCHT was beyond the scope of present study. We also tested the possible inhibition of HCHT by NAG and glucose using 5 μM MU-NAG_2_ as substrate. These monosaccharides were not inhibitory to HCHT at highest concentration (100 mM) tested (data not shown). Using ^14^C-CNWs as substrate, the NAG_2_ inhibition of HCHT was weak with about 80% activity retained in the presence of 2 mM NAG_2_, the highest concentration tested (Fig 5C). With ^14^C-CNW substrate, the NAG_2_ inhibition seemed independent on the concentration of substrate as judged by similar strength and pattern of inhibition observed at 1 mg/mL and 5 mg/mL ^14^C-CNW concentrations (data not shown). With both substrates MU-NAG_2_ and ^14^C-CNWs the strength and the pattern of NAG_2_ inhibition of two HCHT isoforms was identical within error limits.

## Discussion

Human chitinases, HCHT and AMCase are expressed by different cells of immune system and their native substrates are chitin molecules of pathogen origin. This is α-chitin, a chitin with antiparallel orientation of polymer chains in the chitin crystal [59]. β-chitin with parallel orientation of polymer chains has been found only in a few marine organisms and has thus little probability to come into contact with human immune system [60]. However, to date detailed biochemical characterization of HCHT has been made using β-chitin or soluble chitin derivative, chitosan, as substrates [26–30]. Therefore, we made a thorough characterization of HCHT in terms of the mode of action, processivity, binding, and rate constants for the catalysis and dissociation using α-chitin as substrate. Since HCHT has been demonstrated to be active in two isoforms, 50 kDa full-length protein and its 39 kDa truncated version without the C-terminal CBM [19], both were included into the study. The performance of HCHT is discussed in comparison with well characterized chitinases of bacterium *S*. *marcescens* [61].

According to its active site architecture HCHT, is reminiscent to *Sm*ChiA, a processive enzyme moving towards the non-reducing end of chitin chain [43,44]. However, the location of CBM of HCHT is reminiscent to that of *Sm*ChiB, a processive enzyme moving towards the reducing end of chitin chain [44]. When moving towards the non-reducing end, HCHT needs to “drag” its CBM during processive movement. A recent structural study has revealed that the length of the linker peptide is sufficient to enable the location of CBM on both sides of catalytic domain [16] suggesting the possibility to move towards the non-reducing end without “dragging” CBM. The progress curves of the hydrolysis of AA-α-chitin measured here support the reducing-end exo-mode initiation with low processivity (Fig 2). At the same time, comparison of the release of reducing-end label from AA-α-chitin and IRGs generated to reduced α-chitin reveals predominant (95%) use of endo-mode initiation. The corresponding figure reported for *Sm*ChiA is 76% [35]. In parallel with the higher probability of endo-mode initiation, the processivity of HCHT was about 6 fold lower than that of *Sm*ChiA (36.5) measured using the same experimental approach [35]. In comparison with *Sm*ChiA (0.057 1/s for *Sm*ChiA [35]), HCHT had also higher off-rate constant (around 0.4 1/s, Table 1) as judged by the rate of generation of IRGs to reduced α-chitin. Combining the rate constants for catalysis and dissociation provides with the estimates of the P^intr^ values, a parameter that represents the processivity potential of an enzyme [41,42]. Notably HCHT had similar P^app^ and P^intr^ values, a phenomenon characteristic to endo-enzymes [41]. Still, HCHT showed significantly higher processivity and lower off-rate constant than the typical endo-chitinase, *Sm*ChiC (Table 1). Collectively these properties place HCHT in-between processive exo-enzymes and randomly acting non-processive endo-enzymes. The endo-processive character of HCHT may provide explanation to its efficiency in complete degradation of crystalline β-chitin without the aid of synergistic enzyme components like endo-chitinases [29].

Regarding the role of CBM, we note that apart from the *K*_m_ values the two HCHT isoforms had similar kinetic properties. CBM had no effect on the mode of action as revealed by overlapping progress curves (Fig 2) and similar probability of endo-mode initiation (Table 1). Both isoforms had also similar *V*_max_/E_0_ values on different chitin substrates (Table 2) and similar off-rates as well as intrinsic processivities (Table 1). The strength and the pattern of NAG_2_ inhibition of both isoforms was also similar (Fig 5). Although the difference was not prominent, we note that the truncated isoform had somewhat higher apparent processivity on reduced α-chitin (Table 1). Similar trends have been observed in hydrolysis of β-chitin where the processivity (calculated as the NAG_2_/NAG product ratio) values of 11.4 and 7.6 were found for HCHT39 and HCHT50, respectively [29]. The hinge region connecting CBM with catalytic domain contains 31 amino acid residues [16]. This translates into the length of about 10 nm considering the maximum length of the polypeptide chain of approximately 0.34 nm per amino acid residue. Since NAG_2_ is the product of processive hydrolysis and provided with the length of NAG_2_ unit of 1 nm, the average length of processive run of HCHT50 is 5.6 nm (Table 1). The processive run of this length can be achieved just by stretching the hinge region without the need to “drag” CBM. In this context, it is tempting to speculate that the somewhat lower apparent processivity of full-length isoform compared to the truncated version may reflect the constraints posed by the stretching of the hinge region.

The most prominent difference between the two HCHT isoforms is seen in the lower affinity of truncated version for chitin substrates (Table 2). Since both isolated CBM [24,62] and catalytic domain [19] can bind to chitin, the higher affinity of full length enzyme apparently reflects the synergistic interaction between domains in binding. Regarding binding, it is worth noting that about 10% of both isoforms was bound to α-chitin with the active site free for the hydrolysis of MU-NAG_2_ (Fig 3D). Whereas for the full length enzyme the E_bound-FA_ can readily be interpreted as the population of enzyme bound through CBM only, the presence of E_bound-FA_ is less obvious in the case of the CBM-less isoform. The E_bound-FA_ of HCHT39 may represent the population with chitin chain bound to the subsites –6 to –3. Although not identified in HCHT, the presence of surface binding sites cannot be excluded.

It has been proposed that the character of immune response to chitin depends on the particle size (reviewed in [11–13]). This points to a relationship between chitin concentration and HCHT activity, since the surface area available for the binding of HCHT increases with decreasing particle size. The *K*_m_ values of HCHT on α-chitin and CNWs (Table 2) were significantly higher than those measured for *Sm*ChiA in the same experiment system [35]. The low affinity of HCHT to chitin supports the hypothesis that the aim of chitin hydrolysis by HCHT is in the regulation of immune system response rather than in efficient degradation of chitin, since the regulation at substrate level is effective only at substrate concentrations well below *K*_m_. Also, the lysosomal processing of HCHT to remove its CBM further increases the *K*_m_ of the enzyme and in this way may contribute to the sensitivity of the regulation depending on chitin concentration.
